# Novel molecular markers of *Chlamydia pecorum *genetic diversity in the koala (*Phascolarctos cinereus*)

**DOI:** 10.1186/1471-2180-11-77

**Published:** 2011-04-18

**Authors:** James Marsh, Avinash Kollipara, Peter Timms, Adam Polkinghorne

**Affiliations:** 1 Institute of Health and Biomedical Innovation, Queensland University of Technology, Brisbane, Australia

## Abstract

**Background:**

*Chlamydia pecorum *is an obligate intracellular bacterium and the causative agent of reproductive and ocular disease in several animal hosts including koalas, sheep, cattle and goats. *C. pecorum *strains detected in koalas are genetically diverse, raising interesting questions about the origin and transmission of this species within koala hosts. While the *omp*A gene remains the most widely-used target in *C. pecorum *typing studies, it is generally recognised that surface protein encoding genes are not suited for phylogenetic analysis and it is becoming increasingly apparent that the *omp*A gene locus is not congruent with the phylogeny of the *C. pecorum *genome. Using the recently sequenced *C. pecorum *genome sequence (E58), we analysed 10 genes, including *omp*A, to evaluate the use of *omp*A as a molecular marker in the study of koala *C. pecorum *genetic diversity.

**Results:**

Three genes (*inc*A, ORF663, *tar*P) were found to contain sufficient nucleotide diversity and discriminatory power for detailed analysis and were used, with *omp*A, to genotype 24 *C. pecorum *PCR-positive koala samples from four populations. The most robust representation of the phylogeny of these samples was achieved through concatenation of all four gene sequences, enabling the recreation of a "true" phylogenetic signal. *Omp*A and *inc*A were of limited value as fine-detailed genetic markers as they were unable to confer accurate phylogenetic distinctions between samples. On the other hand, the *tar*P and ORF663 genes were identified as useful "neutral" and "contingency" markers respectively, to represent the broad evolutionary history and intra-species genetic diversity of koala *C. pecorum*. Furthermore, the concatenation of *omp*A, *inc*A and ORF663 sequences highlighted the monophyletic nature of koala *C. pecorum *infections by demonstrating a single evolutionary trajectory for koala hosts that is distinct from that seen in non-koala hosts.

**Conclusions:**

While the continued use of *omp*A as a fine-detailed molecular marker for epidemiological analysis appears justified, the *tar*P and ORF663 genes also appear to be valuable markers of phylogenetic or biogeographic divisions at the *C. pecorum *intra-species level. This research has significant implications for future typing studies to understand the phylogeny, genetic diversity, and epidemiology of *C. pecorum *infections in the koala and other animal species.

## Background

*Chlamydia *are obligate intracellular bacterial pathogens that are characterised by a biphasic development cycle, involving the inter-conversion between an extracellular, metabolically inert form (elementary body, EB) and an intracellular, metabolically active form (reticulate body, RB) [1]. With the advent of molecular analyses, the taxonomy of chlamydiae has undergone several revisions [2], with a recent proposal recognising nine species within the *Chlamydia *genus: *C. trachomatis, C. muridarum, C. pneumoniae, C. abortus, C. suis, C. felis, C. psittaci, C. caviae*, and *C. pecorum *[3-5]. For the purpose of this research paper, we will refer to koala *C. pecorum *strains using this proposed nomenclature. While each of these are responsible for a number of disease states in a wide range of animals (including humans), the prevalence and transmission of *C. pneumoniae *and *C. pecorum *throughout Australian koala populations has contributed to a significant decline in koala numbers and remain a critical threat to the koala's continued survival [6-8].

*C. pneumoniae *and *C. pecorum *have been isolated from most koala populations investigated, with *C. pecorum *found to be the most widespread and pathogenic of the two species [7-10]. Notably, *C. pecorum *is also recognised as a pathogen and causative agent of polyarthritis and abortion in sheep and cattle [11]. In the koala, clinical manifestations of *C. pecorum *include ocular infection leading to conjunctival scarring and blindness, respiratory tract infection, urinary tract infection causing incontinence, and genital tract infection potentially leading to infertility [6,7,12-14]. The latter disease signs have been implicated in lowered reproductive rates in wild koala populations in several parts of Australia, highlighting the need to understand this complex host-parasite relationship for the purpose of effective management and control strategies [8].

Questions remain about the evolutionary origin of *C. pecorum *in koalas, given its traditional role as a pathogen of sheep and cattle, and the modes of transmission within and between geographically isolated koala populations. In an attempt to understand these questions, Jackson et al., have previously performed fine-detailed epidemiological surveys of *C. pecorum*-infected koala populations, revealing that *C. pecorum *is genetically very diverse [7]. This analysis was performed on short variable domain IV (VDIV) sequence fragments of the *omp*A gene, encoding the surface-exposed major outer membrane protein (MOMP) which is common to all members of the *Chlamydiaceae *[15]. There are currently eight *omp*A VDIV genotypes that have been identified, following several studies of geographically isolated koala populations in Australia [7,8,14,16,17]. While the majority of these genotypes are apparently confined to the koala host, several identical or near-identical sequences have been found in European sheep and cattle implying the possibility of cross-species transmission events between these hosts [7].

Questions, however, remain regarding the use of *omp*A as a single gene marker of chlamydial diversity. From a phylogenetic perspective, previous studies in other chlamydial species have demonstrated that *omp*A phylogenies are not congruent with the phylogeny of other gene targets, including other membrane proteins [18-20]. Similar observations have also been made for non-koala strains of *C. pecorum *[11,21], indicating that *C. pecorum omp*A gene phylogenies are not congruent with the phylogeny of other targets, nor are they compatible with groupings based on tissue tropisms or pathobiological profiles. Diversifying host immune pressure is hypothesised to cause the *C. pecorum omp*A gene to evolve more rapidly than the rest of the chlamydial genome, rendering it incapable of reflecting the true evolutionary divergence of *C. pecorum *[11].

Until recently, the use of alternate molecular markers for the genetic analysis of koala *C. pecorum *has been limited due to the lack of DNA sequences for this species. However, the recent completion of the currently unpublished *C. pecorum *genome sequence from the E58 type strain is allowing investigation into novel and alternative gene targets. Most notably, Yousef Mohamad et al. recently identified several genes that were potentially useful as *C. pecorum *markers of virulence and pathogenicity [21]. In the current study, we have utilised the *C. pecorum *E58 strain genome sequence in the preliminary characterisation of 10 novel gene targets for the purpose of validating *omp*A as a fine-detailed genetic and phylogenetic marker for *C. pecorum *infections in the koala.

The primary objectives of the present study were to apply our selected genes to (1) a determination of the number of major phylogenetic divisions within koala *C. pecorum *samples obtained from four distinct koala populations; (2) the identification of useful fine-detailed genetic markers to represent these phylogenetic divisions; and (3) a reconstruction of the evolutionary history of lineage divergence between koala and non-koala hosts of *C. pecorum*. Overall, this study identifies useful alternative tools for the future characterisation of koala *C. pecorum *infections. Additionally, we present a preliminary appreciation of the phylogenetic diversity of *C. pecorum *in koala and non-koala hosts, as a prelude to future in-depth multi-locus sequence typing (MLST) studies of the *C. pecorum *phylogeny.

## Methods

### Chlamydial strains and clinical samples

The 'type strain' (MC/MarsBar) utilised for *C. pecorum *gene sequencing and analysis was recently isolated and cultured in our laboratory from a female koala suffering severe genital tract and ocular disease with chronic cystitis. The sample originated from Mount Cotton in South-East Queensland. Swab samples collected from wild koalas were stored at -80°C prior to DNA extraction.

### Selection of candidate molecular marker genes

A total of 10 genes were selected as candidate marker genes, including two housekeeping genes to serve as analysis controls, five membrane proteins and three potential virulence genes. The gene candidates included: 16S rRNA: A housekeeping gene that forms the 16S ribosomal unit; 16S-23S intergenic spacer: A non-transcribed spacer between 16S and 23S ribosomal sequences [22]; *omp*A: Encodes the major outer membrane protein (MOMP) protein, a porin responsible for nutrient transfer, attachment and structural support [23]; *omc*B: A cysteine-rich outer membrane polypeptide with functional, structural, and antigenic properties [24]; *pmp*D: A polymorphic membrane protein and putative autotransporter peptide [25]; *inc*A: Encodes an inclusion membrane protein engaged in the interactions between the chlamydial inclusion and cytosolic components [26]; *cop*N: A virulence-related Type III secretion effector [27]; *tar*P: A translocated actin-recruiting phosphoprotein that recruits actin at the site of internalisation [28]; MACPF: The membrane attach complex/perforin protein and a predicted virulence gene [29]; and ORF663: A hypothetical protein gene whose function is currently unknown [21]. Overall these genes are functionally diverse and are widely distributed around the *C. pecorum *chromosome (data not shown).

### Primers, PCR amplification and sequencing

Primers were primarily based on *C. pecorum *E58 gene sequences. To ensure regions of sufficient sequence conservation were targeted, analyses of homologous gene sequences available from other published chlamydial genomes, including *C. trachomatis, C. pneumoniae, C. caviae, C. felis, C. muridarum*, and *C. abortus *(Table 1), were also performed.

**Table 1:** Chlamydial sequences analysed in this study

Species	Strain	Origin	Host	Pathology	Sequence reference
*C. abortus*	S26/3	Scotland	Sheep	Abortion	[62]
*C. caviae*	GPIC	USA	Guinea Pig	Conjunctivitis	[63]
*C. felis*	Fe/C-56	Japan	Cat	Pneumonia	[64]
*C. muridarum*	Nigg	USA	Mouse	Pneumonia	[65]
*C. pecorum*	824	Scotland	Sheep	Conjunctivitis	[21]
*C. pecorum*	AB10	France	Sheep	Abortion	[21]
*C. pecorum*	AKT	Tunis	Sheep	Abortion	[21]
*C. pecorum*	BE53	England	Cattle	Encephalymylitis	[21]
*C. pecorum*	E58	USA	Cattle	Encephalomylitis	[21]
*C. pecorum*	iB1	France	Sheep	Healthy (faeces)	[21]
*C. pecorum*	iB2	France	Sheep	Healthy (faeces)	[21]
*C. pecorum*	iB3	France	Sheep	Healthy (faeces)	[21]
*C. pecorum*	iB4	France	Sheep	Healthy (faeces)	[21]
*C. pecorum*	iB5	France	Sheep	Healthy (faeces)	[21]
*C. pecorum*	iC2	France	Goat	Healthy (faeces)	[21]
*C. pecorum*	iC3	France	Goat	Healthy (faeces)	[21]
*C. pecorum*	iC4	France	Goat	Healthy (faeces)	[21]
*C. pecorum*	LW679	USA	Sheep	Arthritis	[21]
*C. pecorum*	M14	Morocco	Goat	Abortion	[21]
*C. pecorum*	MC/MarsBar	Australia	Koala	Genital tract infection	(this work)
*C. pecorum*	R69	Ireland	Sheep	Healthy (faeces)	[21]
*C. pecorum*	SBE	England	Cattle	Encephalomylitis	[21]
*C. pecorum*	VB2	France	Sheep	Orchitis	[21]
*C. pecorum*	W73	Ireland	Sheep	Healthy (faeces)	[21]
*C. pneumoniae*	CWL029	USA	Human	Pneumonia	[62]
*C. trachomatis*	A/HAR-13	Saudi Arabia	Human	Conjunctivitis	[63]
*C. trachomatis*	B/Jali20/OT	The Gambia	Human	Conjunctivitis	[62]
*C. trachomatis*	B/TZ1A828/OT	Tanzania	Human	Conjunctivitis	[64]
*C. trachomatis*	D/UW-3/CX	USA	Human	Genital tract infection	[65]
*C. trachomatis*	L2/434/Bu	USA	Human	Bubo	[66]
*C. trachomatis*	L2b/UCH-1/proctitis	England	Human	Proctitis	[66]

Amplification of novel gene sequences from our *C. pecorum *koala type strain began with the addition of 100 ng of semi-purified MC/MarsBar to a PCR mixture containing 1X ThermoPol reaction buffer, 0.2 mM deoxynucleotide triphosphates (Roche), 1 pmol/μL each primer (Sigma; Table 2), and 2 U Vent_R_^® ^DNA polymerase (New England Biolabs). PCR conditions were a single cycle of initial denaturation at 94°C for 2 minutes, 30 cycles of denaturation at 94°C for 1 minute, primer annealing for 1 minute (Table 2), primer extension at 72°C for 2 minutes followed by a final elongation step at 72°C for 10 minutes.

**Table 2:** Genomic region, primers, and melting temperatures for all genes investigated

Gene	Annotation	Primer		Sequence (5' - 3')	**T**_**a**_	Size
*Housekeeping Genes*						

16S rRNA	16S ribosomal subunit		16S-For	CTGAGAATTTGATCTTGG	52°C	1549 bp
			16S-Rev	AAAGGAGGTGATCCAGC		

16S/23S	16S-23S intergenic spacer		Spacer-For	AAGGATAAGGAAAGCTATCA	54°C	225 bp
intergenic spacer			Spacer-Rev	AATTTTTGATCCATGCAAGA		

*Membrane Proteins*						

*omp*A	Outer membrane protein A	1	*omp*A-For	ATGAAAAAACTCTTAAAATCGG	56°C	1170 bp
			*omp*A-Rev	TTAGAATCTGCATTGAGCAG		
		
		2	MJFvd3^a^	GGITG(CT)GCAACTTTAGGIGC	50°C	457 bp
			MJRvd4^a^	CACAAGCTTTTCTGGACTTC		
		
		3	CpeNTVD3^b^	GTTCTTTCTAACGTAGC	46°C	359 bp
			CpeNTVD4^b^	TGAAGAGAAACAATTTG		

*omc*B	Cysteine-rich outer		*omc*B-For	ATGACCAAACTCATCAGAC	54°C	1675 bp
	membrane protein B		*omc*B-Rev	TTAATACACGTGGGTGTTTT		

*pmp*D	Polymorphic membrane		*pmp*D-For	ATGATCAGTCATATACGGAC	56°C	4145 bp
	protein D		*pmp*D-Rev	TTAGAAAATCACGCGTACG		

*inc*A	Inclusion membrane		*inc*A-S-F^c^	TATCGTAATACCAAACCACT	52°C	984 bp
	protein A		*inc*A-S-R^c^	GTGTGAGATGGCTCTTTATG		

*cop*N	*Chlamydia *outer protein N		*cop*N-For	ATGGCAGCTGGAGGGAC	56°C	1191 bp
			*cop*N-Rev	TTATGACCAGGGATAAGGTT		

*Potential Virulence Genes*						

*tar*P	Translocated actin-recruiting phosphoprotein	1	*tar*P-For	ATGACCTCTCCTATTAATGG	56°C	2604 bp
			*tar*P-Rev	CTAGTTAAAATTATCTAAGGTTT		
		
		2	*tar*P-2-For	AAGAACCAACTCTGCATTATGAAGAGG	54°C	768 bp
			*tar*P-2-Rev	AAGAGGTATTCACGCGACTTCCG		

MACPF	Membrane-attack		MAC-For	TTGGCGATTCCTTTTGAAGC	58°C	2346 bp
	complex/perforin protein		MAC-Rev	TTATAAGCACACACTAGGTCT		

ORF663	Hypothetical protein		663-F^c^	AAACAACTGCACCGCTCTCT	55°C	1167 bp
			663-R^c^	GAAGGACTTTCTGGGGGAAG		

^1^primers used for initial sequencing of full-length gene from MC/MarsBar/UGT type strain; ^2/3 ^primers used for second-stage sequencing from koala populations for further analysis; ^a^primers designed by [7]; ^b^primers designed by [10]; ^c^primers designed by [26].

Due to the low quality and quantity of template from the koala clinical samples, an alternate PCR protocol was adopted which was optimised for higher specificity and sensitivity. This was achieved by the addition of 5 μL of DNA extracted from *C. pecorum*-positive swab samples to a PCR mixture containing 1X AmpliTaq Gold 360 10 × buffer, 0.2 mM of each deoxynucleotide triphosphate (Applied Biosystems), 1 pmol/μL each primer (Sigma; Table 2), and 1 U AmpliTaq Gold 360 DNA polymerase™ (Applied Biosystems). PCR conditions were a single cycle of initial denaturation at 95°C for 10 minutes, 45 cycles of denaturation at 95°C for 1 minute, primer annealing for 1 minute (Table 2), primer extension at 72°C for 1 minute, followed by a final elongation step at 72°C for 7 minutes.

PCR products for both assays were separated by gel electrophoresis and visualised using a UV transmilluminator. Negative controls (dH_2_O) were included in each amplification round to control for PCR contamination. PCR products were purified with an Invitrogen PureLink™ PCR purification kit and sent to the Australian Genome Research Facility (AGRF) for sequencing using the Sanger dideoxy method [30]. Gene sequence names from each *C. pecorum *positive sample were derived from the population from which the koala originated and the ID name assigned by the veterinarians (i.e. 'Bre/Ned' = Brendale population; animal name 'Ned').

### Sequence and statistical analysis

Alignments for each sequenced gene were produced using ClustalW [31] and RevTrans [32] was used to reverse-translate all alignments. Non-coding genes were aligned based on their nucleotide sequence.

The software package DnaSP 5.0 [33] was used to analyse the extent of sequence variation by calculating the number of polymorphic and parsimony-informative sites, the average nucleotide diversity (*p*-distance) and Tajima's test for neutrality (*D*-value). The Molecular Evolutionary Genetics Analysis (MEGA) [34] software package was used to calculate the number of synonymous and non-synonymous sites and subsequent d*N*/d*S *ratio using the Nei-Gojobori method [35]. The discrimination index (D.I.), based on Simpson's index of diversity [36], was calculated to determine the differentiating and discriminatory capacity of each gene:

where *D *= index of discrimination, *N *= number of strains in the sample, and *n*_i _= number of strains in group i. The index ranges from 0 to 1, with a value close to 0 indicating low genetic diversity and a value close to 1 indicating high genetic diversity [36]. Calculation of the D.I. requires at least three nucleotide sequences for analysis.

### Criteria for identifying genetic markers

In order to select the most appropriate candidate genes for further investigation, a shortlist of three genes, ORF663, *inc*A and *tar*P (in addition to *omp*A), were selected based on their application in previous *C. pecorum *typing studies [21], in addition to several empirical criterions: The average proportion of nucleotide distances (*p*-distance) should be ≥ 0.02 before intra-species differentiation may be attempted [37,38], which can be calculated from an alignment containing two or more sequences [39,40]. Furthermore, both highly constrained, slowly-changing molecular markers and highly variable genes under diversifying selection each have their advantages, disadvantages, and advocates [41], implying the importance of selecting genes under both positive and negative selection. Finally, the discrimination index (D.I.) for candidate markers should be > 0.50, which is suggested to be sufficient discriminatory power for adequate differentiation of bacteria beyond the species level [42-44].

### Koala populations, swab collection and processing

Four distinct Australian koala populations were studied: East Coomera, Brendale, Narangba, and Pine Creek. The East Coomera population is located in South-East Queensland, approximately 54 km south of Brisbane and is comprised of approximately 500 koalas located in a 1716 ha area of cleared lands with isolated trees and small patches of native vegetation. The Brendale and Narangba populations are located among residential developments on the outskirts of Brisbane and are separated by a busy highway. The Pine Creek population is situated 20 km south of Coffs Harbour, New South Wales and consists of approximately 6400 ha of coastal eucalypt forest interspersed with pockets of rainforest, pasture and freehold incursions. The Pine Creek population was previously surveyed and was found to have 52% *C. pecorum *PCR positivity amongst animals screened [9].

A total of 295 ocular and urogenital swabs were collected from 80 koalas within the four populations. Ethics approval for the collection of swab samples from koalas was considered and provided by the QUT Animal Research Ethics Committee (Approval number 0900000267).

For each sample, vials containing swabs and sucrose phosphate glutamate (SPG) transport media were vortexed for 30 seconds to release chlamydial bodies from the swab. 1 mL was transferred to a 1.5 mL eppendorf tube and centrifuged at 13,000 × g for 30 minutes to pellet the sample. Following removal of the supernatant, the pellet was resuspended in 50 μL of SPG transport media and heated to 100°C for 2 minutes to release the DNA. Chlamydial DNA was then extracted using the tissue protocol of the QIAamp DNA kit (Qiagen).

### *C. pecorum*-specific diagnostic quantitative real-time PCR

A total of 82 swabs from urogenital and ocular sites of the Narangba, Brendale, Pine Creek, and East Coomera koalas (65 animals) were screened for the presence of *C. pecorum *using a diagnostic quantitative real-time PCR (RT-PCR) targeting a 204 bp fragment of the 16S rRNA gene.

The RT-PCR assay involved the addition of 3 μL of chlamydial DNA to a PCR mixture containing 1 × Faststart Taq DNA polymerase reaction buffer (Roche), 0.2 mM deoxynucleotide triphosphates (Roche), 10 μM primers (RT-Pec.sp-F: 5'-AGTCGAACGGAATAATGGCT-3', RT-Pec.sp-R: 5'-CCAACAAGCTGATATCCCAC-3'; Sigma), 0.25 U/μL Faststart Taq DNA polymerase (Roche), and 1X SensiMix*Plus *SYBR green (Quantace). All samples were assayed in triplicate. The MC/MarsBar type strain served as a positive control while dH_2_O was used as the negative control. PCR conditions were an initial denaturation of 94°C for 3 minutes, 40 cycles of denaturation at 94°C for 15 seconds, primer annealing at 57°C for 30 seconds, and DNA elongation at 72°C for 25 seconds. This was followed by a melting step from 70-90°C. Equal numbers of *C. pecorum *positive samples (n = 6) were randomly selected for further PCR amplification, sequencing, and analysis.

While *inc*A and ORF663 were amplified and sequenced as full-length genes, smaller fragments of *omp*A and *tar*P were used for analysis. These included a 465 bp fragment of *omp*A that comprises the highly variable VD III and IV regions which were previously targeted in a range of phylogenetic and fine-detailed epidemiological studies [11,21] and a 726 bp highly polymorphic fragment of the *tar*P gene.

### Phylogenetic analysis

Phylogenetic reconstructions were performed under both distance and maximum-parsimony frameworks. Distance analyses were performed using the neighbour-joining algorithm and the Tamura-Nei model of molecular evolution as implemented in MEGA. Maximum parsimony analyses were conducted by using the tree-bisection and reconnection method of branch swapping and the heuristic search algorithm of PAUP* version 4.0b. Relative support for individual nodes was assessed by nonparametric bootstrapping, with 1000 replications of the data. The pairwise-deletion option was chosen to remove all sites containing missing data or alignment gaps from all distance estimations. Optimisation of the branch lengths was done by using the maximum-likelihood method (using Modeltest to define the evolutionary parameters [45]), subject to the constraint that all sampled sequences were contemporary (i.e., molecular clock was enforced). All rooted trees were constructed with mid-point rooting to facilitate genotypic comparisons of the outer topologies.

### Genotypic analysis

The ability of each of the shortlisted genes to define specific genotypes within the koala populations was assessed, based on the nucleotide dissimilarity of sequences. To facilitate comparisons with previous research on koala *C. pecorum *infections, a similar genotyping approach was adopted where nucleotide dissimilarity > 1% (based on multiple sequence alignments of all koala strains for each gene) results in a new genotype [7,8,46]

### Recombination

Recombination Detection Program (RDP) was used to test aligned sequences for recombination. This package utilises six published methods found to be sensitive for the identification of recombination and to yield the fewest false-positive findings [19]. The six methods are: RDP [47], GENECONV [48], Bootscan [49], MaxChi [50], Chimaera [51], and SiScan [52]. Different tests are applied to aligned sequences by each method to detect potentially recombinant regions [19]. The null hypothesis is clonality, i.e., that the pattern of sequence variation among the aligned sequences shows no indication of recombination [19]. Recombination was deemed to occur in a locus if clonality was rejected by three or more tests at a significance level of *P *< 0.001 [19].

### GenBank accession numbers of novel sequences

All novel *C. pecorum *sequences characterised in this study were submitted to GenBank and are available according to accession numbers HQ457440 to HQ457545.

## Results

### PCR amplification and sequence analysis of 10 candidate molecular markers from the koala *C. pecorum *type strain (MC/MarsBar)

Successful PCR amplification of each of the 10 gene loci was achieved using the primers and conditions outlined in Table 2. Analysis of the gene sequences for the selected targets is summarised in Table 3. The *omp*A, *inc*A, *cop*N, and ORF663 gene sequences were analysed in conjunction with previously published *C. pecorum *data (Table 1), while the 16S rRNA, 16S/23S intergenic spacer, *omc*B, *pmp*D, *tar*P, and MACPF genes were compared with the E58 reference strain as no other data is currently available for these genes.

**Table 3:** Summary of nucleotide sequence variation between the MC/Mars Bar koala *C. pecorum *type strain and non-koala *C. pecorum *strains in sampled regions of the *C. pecorum *genome

Group and locus	N	Size (bp)	AlleleNo.	Δnt	%nt	π	Δrep	%rep	Δnon-rep	%non-rep	d*N*/d*S*	D	Pars	D.I.
*Housekeeping Genes*														

16S rRNA	2	1549	2	2	0.130	0.001	N/A	N/A	N/A	N/A	N/A	N/A	0	N/A
16S/23S intergenic spacer	2	225	1	0	0.000	0	N/A	N/A	N/A	N/A	N/A	N/A	0	N/A

*Membrane Proteins*														

*omp*A	20	1170	13	122	10.430	0.162	72	59.020	21	17.210	0.170	1.734	111	0.910
*omc*B	2	1675	2	8	0.420	0.004	7	87.500	1	12.500	2.150	N/A	0	N/A
*pmp*D	2	4145	2	20	0.480	0.005	13	65.000	5	25.000	0.670	N/A	0	N/A
*inc*A	20	984	17	116	11.790	0.656	78	67.240	19	16.380	1.540	0.703	59	0.980
*cop*N	20	1191	9	9	0.760	0.008	9	55.560	5	44.440	0.550	1.163	7	0.880

*Potential Virulence Genes*														

*tar*P	2	2604	2	56	2.150	0.029	37	66.070	19	33.903	0.660	N/A	0	N/A
MACPF	2	2346	2	7	0.300	0.003	5	71.430	2	28.570	0.730	N/A	0	N/A
ORF663	20	552	18	66	11.960	0.741	29	43.940	23	34.850	1.350	0.381	48	0.980

N: no. of *C. pecorum *sequences analysed; Allele no.: no. of unique sequences according to gene; Δnt: number of polymorphic nucleotide sites; %nt: percent nucleotide sites polymorphic; π: average *p*-distance at all sites; Δrep: number of polymorphic sites resulting in an amino acid replacement; %rep: percent sites with replacement; Δnon-rep: number of polymorphic sites not resulting in an amino acid replacement (synonymous changes); %non-rep: percent sites with non-replacement; d*N*/d*S*: ratio of the number of non-synonymous (d*N*) to synonymous (d*S*) substitutions per site; D: Tajima's test for neutrality; Pars: parsimony-informative sites; D.I.: discrimination index; D: Tajima's test for neutrality.

In total, 16244 bp of data was analysed which represents 1.62% of the complete *C. pecorum *genome. The two housekeeping and non-coding genes, 16S rRNA and 16S/23S intergenic spacer, were sampled to provide a counterpoint to the coding sequence data and represent genes under stabilising selection. Across a total of 3548 bp of data from these two genes, only two SNPs were observed (0.13%).

Analysis of *omp*A revealed a significantly higher level of polymorphisms (122), which equated to 10.43% of the 1170 bp gene and a mean diversity of 0.162. Both *inc*A and ORF663, while possessing fewer individual polymorphisms than *omp*A (116 and 66 respectively), exhibited a higher percentage of nucleotide diversity at 11.79% and 11.96% respectively. This equated to a mean diversity of 0.656 for *inc*A and 0.741 for ORF663. Together *omp*A, *inc*A, and ORF663 were the most divergent genes out the 10 investigated. The remaining candidates were significantly more conserved with a five-fold reduction in nucleotide diversity. *Tar*P exhibited 56 individual polymorphic sites out of 2604 bp (2.15%) for an average diversity score of 0.029, while MACPF was the most conserved of the coding genes investigated with only seven polymorphic sites (0.30%), resulting in a mean diversity of 0.003.

Within *omp*A, there were 72 mutations leading to a change in amino acid (non-synonymous mutations), representing 59.02% of the total nucleotide diversity for this locus. The d*N*/d*S *ratio for *omp*A was therefore 0.17, which correlates with the D-value of 1.73 indicating *omp*A's considerable deviation from neutrality and tendency for negative selection. Interestingly, out of all eight coding genes investigated, *omp*A maintained the lowest percentage of non-synonymous mutations and therefore the lowest d*N*/d*S *ratio. The *omc*B gene represented the opposite end of the scale with 87.5% of mutations leading to an amino acid replacement with a d*N*/d*S *ratio of 2.15.

The number of parsimony-informative sites and the discrimination index (D.I.) were calculated to enable each locus to be graded according to their discriminatory capacity, however, it is important to note that the estimates for both tests remain limited due to the mutual requirement for more than two sequences for analysis. Nevertheless, *omp*A had the most parsimony-informative sites (111 sites), approximately twice as many as *inc*A (59 sites). These results were slightly altered when considering the D.I. values as both *inc*A and ORF663 scored the highest (both 0.98), while *omp*A remained at 0.91 and *cop*N at 0.88.

### The *omp*A, *inc*A, *tar*P, and ORF663 genes are potentially useful intra-species molecular markers of koala *C. pecorum *infections

Based on the defined criteria for selecting fine-detailed molecular markers (see Materials and Methods), the *omc*B, *pmp*D, MACPF, and *cop*N genes had insufficient mean diversity and were not selected for further analysis. Conversely, the *omp*A, *tar*P, *inc*A, and ORF663 genes were able to satisfy this criterion and in addition, represent loci under diverse selection processes. Three of these four genes also offered useful D.I. values, while the unavailability of additional sequence data for *tar*P prevented its calculation. Nevertheless, *tar*P's adequate mean diversity and tendency for negative selection provided an important counterpoint to the highly divergent, positively-selected *inc*A and ORF663 genes.

### Phylogenetic analysis of the *omp*A, *inc*A, *tar*P, and ORF663 genes from clinical samples

The phylogenetic analysis of our four targeted genes was prefaced with an evaluation of the mean genetic diversity for each locus based solely on the koala populations, in comparison with the data generated for non-koala hosts (Table 3). We observed a decreased level of mean diversity for *omp*A (*p *= 0.096), ORF663 (*p *= 0.065), and *inc*A (*p *= 0.016), which is anticipated given the expected contrast between the genetic variation present in our koala populations and the global samples of *C. pecorum *from multiple animal hosts. Interestingly, the *tar*P gene produced a comparable figure of *p *= 0.028. These results are significant from a global *C. pecorum *genetic diversity perspective, but this remains outside the scope of this study. In the context of the current study, this data importantly demonstrated that the *inc*A value of *p *= 0.016 for the koala populations is below the *p *= 0.02 threshold required for intra-species differentiation.

**Figure 1:**
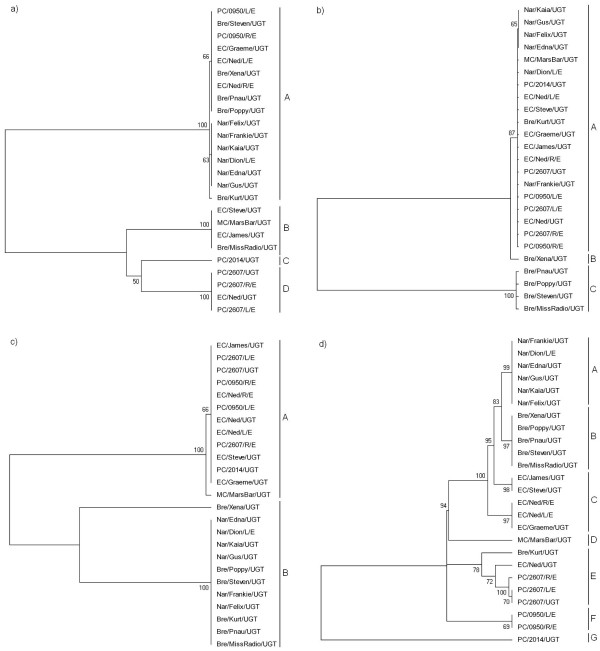
**Mid-point rooted phylogenetic trees based on each of the four candidate genes**. Inferred by the neighbour-joining method with bootstrapping support (1000 replicates). a) *omp*A; b) *inc*A; c) *tar*P; d) ORF663.

Examination of the resulting phylogenetic trees revealed a level of resolution that was consistent with the corresponding gene's mean nucleotide diversity within the koala strains (Figure 1). Between each of the four trees there remained a consistent dissimilarity of branching orders, each with varying degrees of bootstrap support. Overall, there was a tendency for *omp*A and ORF663 to separate the Narangba and Brendale populations from the East Coomera and Pine Creek populations, while the *tar*P phylogenetic tree provided the most robust evidence for this distinction (Figure 1). The *inc*A tree revealed less resolution between *C. pecorum *positive samples, correlating with its low level of mean sequence diversity and discriminatory power (Table 3).

**Figure 2:**
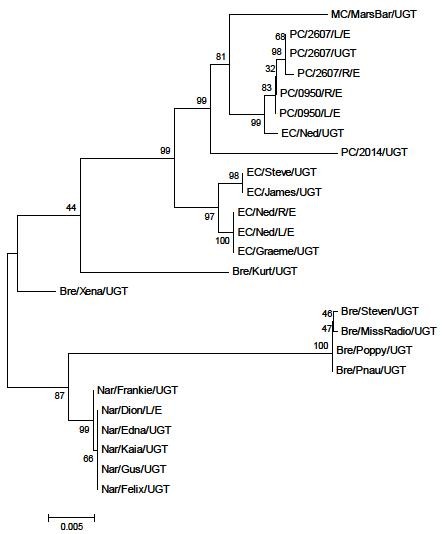
**Phylogenetic tree from concatenated sequences of *omp*A, *inc*A, ORF663, and *tar*P from all koala populations**. Mid-point rooted and inferred by the neighbour-joining method with bootstrapping support (1000 replicates).

To create a more comprehensive data set to permit more robust phylogenetic inferences, sequences for each of the four genes were concatenated and used in the construction of an additional phylogenetic tree (Figure 2). This tree produced largely similar groupings to those described above with the separation of the Narangba and Brendale populations from the Pine Creek and East Coomera populations, as well as the isolation of the more divergent *C. pecorum *positive samples from their respective populations. To test whether the phylogeny resulting from the concatenated sequence was biased by a single locus, a subset of trees was built using the concatenated data with each region omitted. This resulted in no perturbation of the tree topology (data not shown).

**Figure 3:**
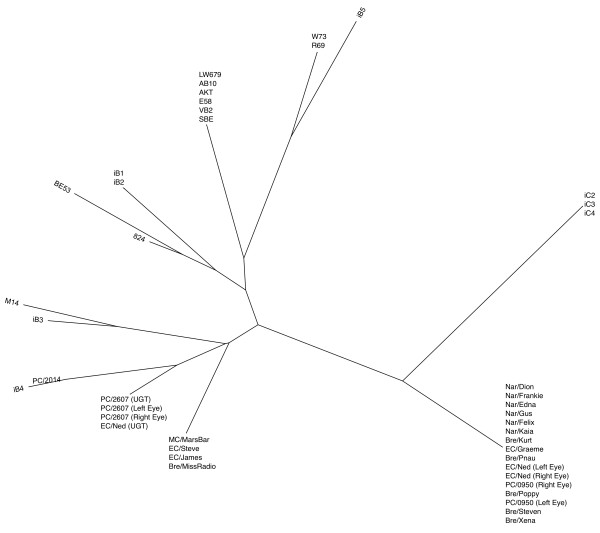
**Phylogenetic tree of *omp*A sequences from koala *C. pecorum *isolates, with previously published sequence information**. Unrooted; inferred by the neighbour-joining method with bootstrapping support (1000 replicates).

**Figure 4:**
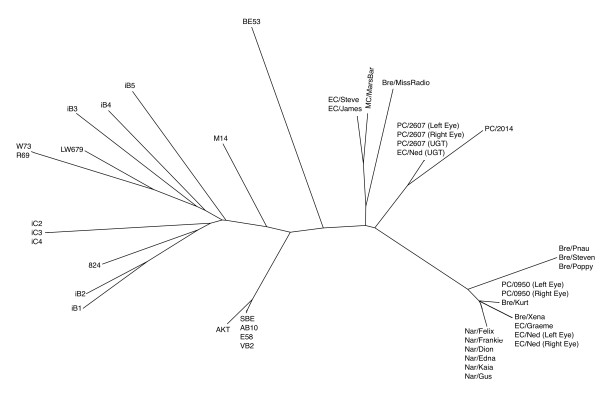
**Phylogenetic tree of the koala *C. pecorum *isolates sequenced, with previously published sequence information**. Unrooted; constructed using concatenated sequences of *omp*A, *inc*A, and ORF663 using the neighbour-joining method with bootstrapping support (1000 replicates).

In addition, a phylogenetic analysis was performed to examine the relationship between the koala *C. pecorum *samples analysed in this study, and other previously sequenced strains from non-koala hosts (Table 1). Initially a tree was constructed using only *omp*A data (Figure 3) which clearly shows the koala *C. pecorum *sequences grouping with sheep and/or cattle strains rather than with each other. Subsequently, the sequence data for *omp*A, *inc*A, and ORF663 were concatenated and a single phylogenetic tree constructed. While there was no visible relationship between geography or body site of infection, there was a clear separation between the koala and non-koala strains (Figure 4). As ancestral relationships are not being inferred between the koala and non-koala hosts, unrooted phylogenetic trees were used to illustrate this data.

### Genotypic analysis of the *omp*A, *inc*A, *tar*P, and ORF663 genes

To highlight the discriminatory power of *omp*A, *inc*A, *tar*P, and ORF663, *C. pecorum*-specific genotypes were established based on their level of nucleotide dissimilarity and aligned with the phylogenetic gene trees outlined above (Figure 1). The *omp*A gene was able to separate the koala samples into four genotypes, the *inc*A gene produced three genotypes, the *tar*P gene separated the clinical samples into two genotypes, while ORF663 was able to discriminate between seven distinct genotypes.

### Recombination

Each of the four shortlisted genes (*omp*A, *inc*A, ORF663, *tar*P) was tested for evidence of recombination by the RDP. All sequences were found to deviate from clonality by all six recombination tests (*P *< 0.001), which is consistent with previous reports regarding *omp*A and ORF663 [19,53].

## Discussion

The current study revealed three novel and significant characteristics of the evolution and genetic diversity of *C. pecorum *infections in the koala: (1) the *omp*A gene has a phylogenetic history that is congruent with other gene targets in the *C. pecorum *genome, yet is phylogenetically-insufficient for use as a single gene marker; (2) the *tar*P and ORF663 genes are potentially useful in representing *C. pecorum *genomic diversity and evolution, and (3) koala *C. pecorum *infections appear to be monophyletic, possibly suggesting a limited number of cross-host transmission events between koalas and non-koala hosts.

The *omp*A gene is one of the most polymorphic genes across all *Chlamydia *species [23] and as a result, was previously selected as the molecular marker of choice in epidemiological and genotyping studies of *C. pecorum *infections of the koala. This increased nucleotide diversity is reported to be due to the antigenicity of MOMP and the selective pressure of the host's immune response [54]. Early *C. trachomatis *studies and more recent *C. pecorum *studies suggested that the phylogenetic categorisation of the *omp*A gene is not concordant with pathobiotypes, tissue tropisms, or the evolution of the genome as a whole [7,11,18,20,21]. Based on these findings, the use of *omp*A gene as a molecular marker of koala *C. pecorum *genetic diversity also required re-evaluation.

Assumptions on the validity of *omp*A as a genetic marker for koala *C. pecorum *strains must be preceded by an appreciation of the koala *C. pecorum *phylogeny. Without in-depth MLST studies to determine the true *C. pecorum *phylogeny, this study applied our four genes of interest (*omp*A, *inc*A, ORF663 and *tarp*), to a multi-locus approach to phylogeny in an effort to recreate the most accurate phylogenetic signal (Figure 2) using single gene targets. Some level of phylogenetic discordance is expected between these genes given their diverse metabolic function, chromosomal location, possibility for evolutionary rate heterogeneity and the susceptibility of all four genes to recombination events. However, this multi-locus method benefits from a "majority rule" approach by allowing the amplification of congruous phylogenetic information while reducing the effects of phylogenetic "noise". In addition, the equalisation of outer branch lengths serves to resolve minor phylogenetic inconsistencies. Together, this results in a more accurate phylogeny than that inferred from a single gene [55,56]. There was no perturbation of the tree topology when each gene was sequentially omitted from analysis, alleviating concerns that individual genes may dominate and sweep the phylogenetic signal. It is expected that the systematic addition of further gene data will continue to produce a more refined and resolute phylogeny, however we suggest that the phylogenetic tree using concatenated sequences of *omp*A, *inc*A, ORF663, and *tar*P provides a preliminary and useful indication of the true phylogenetic relationship between these koala *C. pecorum *samples and a prelude to future MLST and phylogenetic studies.

The phylogenetic tree generated from concatenated data clearly defines two distinct lineages between the four populations investigated: (1) the Pine Creek and East Coomera populations (separated by ~500 kms), and (2) the Narangba and Brendale populations (separated by ~5 kms), while each lineage is further subdivided into two clades, each representing an individual population. From an evolutionary standpoint, this phylogenetic reconstruction appears valid. For example, it is clear that the Brendale and Narangba populations remain geographically (and genetically) similar, as do the East Coomera and Pine Creek populations, albeit to a lesser degree. The genetic diversity and uniqueness of geographically isolated *C. pecorum *strains is presumably the result of disturbances to koala population distribution and structure from land clearing and urban pressure over the last 200 years of European settlement, leading to the formation of isolated koala colonies in which *C. pecorum *strains continue to undergo local selection and adaptation. The question that remains is how effective are the four shortlisted genes in abbreviating this vast phylogenetic information for epidemiological study?

Beginning with *omp*A, previous *C. pecorum *studies suggest that this gene is reflective of the overall evolution of the *C. pecorum *genome [7,23], however these studies are based on broad comparisons between chlamydial species and do not represent evolutionary lineages on an intra-species level. Alternatively, intra-species *C. trachomatis *studies have indicated that the *omp*A locus differs from other regions of its genome [19]. The results of the present study illustrate a tendency for the phylogenetic topology of the *omp*A gene to separate the Narangba/Brendale populations from the Pine Creek/East Coomera populations while other, more divergent strains do not cluster according to their respective population. This data would appear to correlate with previous *C. pecorum *fine-detailed epidemiological studies where it was concluded, using the *omp*A gene, that an association between the site of koala capture and the genotype of its resident *C. pecorum *strain usually exists, while some genotypes were distributed widely into different geographic areas [7]. The phylogenetic divisions offered by the tree using concatenated sequences, however, clearly show that regions of the *omp*A gene are actively contributing to a misinterpretation of the "true" phylogenetic signal. This observation supports previous conclusions that *omp*A is ineffective as a genome-representative marker. It is therefore suggested that while the *omp*A gene continues to be a useful fine-detailed comparative marker, it remains suboptimal for any phylogenetic, evolutionary and/or biogeographic analysis. Both the *tar*P and ORF663 genes, conversely, are appealing alternatives to *omp*A.

The *tar*P gene encodes the translocated actin-recruiting phosphoprotein [57] which has important virulent functions involved in the attachment of the chlamydial elementary body to the host cell [28]. The *tar*P gene's tendency for negative selection and relatively low mean nucleotide diversity reinforces its important biological role in the chlamydial cell and typifies a gene that changes slowly enough to make it useful as an evolutionary chronometer [41]. Recent *C. trachomatis *studies have suggested that the full-length *tar*P gene, based on the inverse relationship between the number of tyrosine repeats and the number of actin-binding domains, can be correlated with clinical phenotype [58], highlighting its potential as a useful genetic marker.

The koala *C. pecorum tar*P gene phylogenetic tree produced two distinct clades which, interestingly, revealed a clear separation between the Brendale and Narangba isolates and the Pine Creek and East Coomera isolates. Initially, there appeared to be no distinction between ocular and urogenital sites of infection, however upon further inspection, it was clear that (with the exception of Nar/Dion (Left Eye)), all the ocular isolates remained confined to one phylogenetic clade (among seven urogenital isolates) which are distinct from the remaining urogenital isolates. Importantly, this ocular "outlier" (Nar/Dion (Left Eye)) retains 100% nucleotide similarity with the remaining isolates within the Narangba population, all of which were isolated from urogenital sites of infection. Coupled with the fact that isolate 'Ned' from the East Coomera population harbours genetically distinct ocular and urogenital isolates of *C. pecorum*, this suggests that high rates of transmission within these confined koala populations may contribute to the transfer of *C. pecorum *from one body site to another and that the site of detection may not be the original niche of the strain [58]. It appears that the *tar*P gene has potential as a phenotypic-dependent marker, however, importantly, further investigation is required that utilises the full-length *tar*P gene (in conjunction with wider geographic sampling) to properly determine its true potential.

From a full genome evolutionary standpoint, the separation of the Brendale/Narangba populations from the Pine Creek/East Coomera populations is a distinction that is clearly mirrored in the overall phylogenetic analysis using concatenated data. This suggests that *tar*P, although having a relatively low rate of substitution, is capable of more accurately and specifically differentiating koala strains according to geography than *omp*A and ORF663, albeit with reduced resolution. For these reasons, *tar*P also appears promising as an evolutionary indicator and may be classified as a "neutral marker", characterised by its selective constraints yet ability to reflect sequence diversity between koala populations that are geographically separate [59]. However, as a "neutral marker", the *tar*P gene remains less useful when estimating a population's adaptive potential or local population divergence.

ORF663 encodes a hypothetical protein and includes a 15 nucleotide variant coding tandem repeat (CTR) region that putatively associates it with a virulence-related role. Interestingly, this gene has not been identified in any other chlamydial species and BLAST search reveals no similarities to any other sequences in the database. The *C. pecorum *ORF663 gene was the most polymorphic gene among all investigated and represents a locus under considerable positive selection. Using this gene, we were able to observe the most distinctions between strains by identifying seven separate genotypes. These genotypes highlight the considerable discriminatory capacity of ORF663 which correlates with (while extending) the divisions made by *omp*A and *tarP*, by isolating the Narangba and Brendale populations into their own genotypes while separating the more heterogeneous Pine Creek and East Coomera populations into multiple genotypes. Where the *tar*P gene represents a neutral marker that assumes isolates within a population are equally related to each other, ORF663 can be considered a "divergence-based" or "contingency" marker that is capable of characterising diversity both within and between populations for fine-detailed epidemiological study.

The value of the marker genes identified in this study was extended to consider the genetic diversity between *C. pecorum *infections in koalas and non-koala hosts. Previous research has suggested that, supported by *omp*A VD3/4 sequence data, *C. pecorum *is a polyphyletic organism in Australian koala populations. This hypothesis originated from the similarity of one or two koala *omp*A genotypes to European bovine isolates of *C. pecorum *[7,11] and based on this data, a model was proposed whereby koalas obtained *C. pecorum *infections as a result of a series of cross-species transmission events from sheep and/or cattle [7,8,11,60]. While similar results were obtained using *omp*A data in this study (Figure 3), the phylogenetic analysis has already suggested in inadequacy of the *omp*A gene alone in representing *C. pecorum*'s true evolutionary course within koala populations. Indeed, both this and previous studies utilised a 465 bp fragment of the *omp*A locus (VD 3/4) which, while containing the majority of *omp*A's nucleotide variation, would remain largely insufficient to describe the extensive genetic diversity that has accumulated in global isolates of *C. pecorum*.

Consequently, we prepared an unrooted phylogenetic tree from the concatenation of *inc*A, *omp*A, and ORF663 sequences, revealing a surprising alternative picture that clearly distinguishes koala *C. pecorum *strains from non-koala hosts (Figure 4). This distinction is further supported by the noticeable difference in branch lengths between koala *C. pecorum *sequences and non-koala hosts, suggesting that as a whole, koala strains are much more closely related to each other than to other non-koala host strains. This result is significant as it may be an example of an alternate evolutionary model in which koalas obtained *C. pecorum *as a result of a limited number of cross-host transmission events in the past and have subsequently evolved along an evolutionary trajectory that is distinct from that seen in sheep and cattle isolates. This result also reinforces the benefit and efficacy of applying more phylogenetically-robust data (the concatenation of three congruent genes) to the epidemiological study of *C. pecorum *infections, both in koala and non-koala hosts. It must be noted however, that this remains a cautionary finding. Without *omp*A, *inc*A, and ORF663 nucleotide sequences from Australian sheep and cattle isolates it remains impossible to truly establish a compelling cross-host transmission hypothesis for koala isolates. Nevertheless, this data cannot be completely discounted and functions as preliminary insight into the genetic diversity of koala isolates of *C. pecorum*.

## Conclusions

The findings of this study have highlighted the opportunities and drawbacks of estimating phylogenetic relationships from multiple independent datasets [61]. A concatenation approach to phylogenetic inference appears promising, however a true evolutionary reconstruction of the *C. pecorum *lineage may require a rigorous MLST approach that incorporates genetic data from several more independent loci and extensive geographic sampling.

It is clear that the *omp*A gene is distorted by technical and biological interference rendering it incapable of representing true phylogenetic divisions as a molecular marker, yet it remains useful as a fine-detailed, cost-effective, comparative marker for fine-detailed epidemiological investigation of large numbers of koala *C. pecorum *positive samples. Alternatively, the *tar*P gene's ability as a "neutral marker" to provide a "bird's-eye-view" on higher levels of evolutionary divergence between koala populations and ORF663's opportunities as a contingency marker are promising for future phylogenetic studies in the koala.

While three out of our four shortlisted genes (including *omp*A) proved to be effective gene markers, *inc*A was ultimately deemed to be the least effective and was discarded from further analysis. However, the significant discrepancy noted between the mean diversity of *inc*A from koala and non-koala hosts (as well as ORF663) invites intriguing questions regarding the genetic diversity of *C. pecorum *beyond the koala host which, while outside the scope of this study, will be important in subsequent research in this area.

Although this study focussed on a mere 10 genes in the *C. pecorum *genome, it successfully challenged *omp*A as a molecular marker and provided an important opportunity to review previous knowledge on the genetic diversity of *C. pecorum *in Australian koala populations. The availability of the complete E58 *C. pecorum *genome sequence and, eventually, a koala *C. pecorum *genome, will facilitate the characterisation of additional genes and promote further analyses of genomic variation to support comprehensive surveys of lineage prevalence within and between koala populations. Until then, the data described here provides a solid foundation for this subsequent research by highlighting a robust measurement tool for koala *C. pecorum *infections and presents a compelling depiction of their phylogenetic relationships. This application will have importance for our ability to successfully map, control and manage diseased populations of this dwindling native icon.

## Authors' contributions

JM carried out the laboratory work, performed all sequence, phylogenetic and statistical analyses, and drafted the manuscript. AK performed the processing of koala swabs, PCR screening and *omp*A sequencing of *C. pecorum*-positive samples. PT and AP conceived the study, participated in its design and coordination and assisted in drafting the manuscript. All authors read and approved the final manuscript.
